# Hijacking emergency granulopoiesis: Neutrophil ontogeny and reprogramming in cancer

**DOI:** 10.1002/1878-0261.70241

**Published:** 2026-03-19

**Authors:** Gabriela Marinescu, Yi Feng

**Affiliations:** ^1^ Centre for Inflammation Research, Institute for Regeneration and Repair University of Edinburgh UK; ^2^ Cancer Research UK Scotland Centre, Institute of Genetics and Cancer University of Edinburgh UK

**Keywords:** neutrophil ontogeny, neutrophil plasticity, tumour microenvironment, tumour‐induced granulopoiesis

## Abstract

Neutrophils are abundant innate immune cells with remarkable plasticity, capable of exerting both antitumour and protumour functions. Beyond their local roles in the tumour microenvironment, recent studies highlight tumour‐induced granulopoiesis as a systemic process by which cancers rewire haematopoiesis to expand immature neutrophils with immunosuppressive and tumour‐promoting activity. Sustained by tumour‐derived cytokines, chemokines and alarmins, tumour‐induced granulopoiesis activates developmental programmes such as STAT3–C/EBPβ and RORC1, driving persistent neutrophilia and systemic immune suppression. Here, we review neutrophil maturation and heterogeneity, their dual roles in tumour initiation and progression, and the emerging recognition of tumour‐induced granulopoiesis as a critical axis of tumour–host interaction with clinical and therapeutic implications.

## Graphical abstract

Neutrophils are highly plastic innate immune cells; their functions in cancer extend beyond the tumour microenvironment. This Review summarises current understanding of neutrophil maturation and heterogeneity and highlights tumour‐induced granulopoiesis as a systemic programme that expands immature, immunosuppressive neutrophils via tumour‐derived inflammatory cues. We discuss key regulatory pathways and emerging biomarker and therapeutic opportunities.

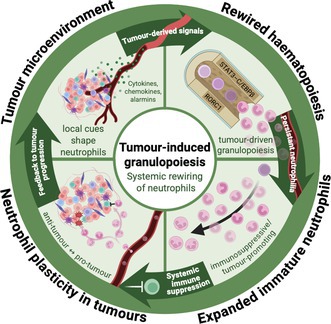

**Abbreviations**
ARNTL: Aryl hydrocarbon receptor nuclear translocator‐like protein 1
BM: Bone marrow
BMAL1: Basic helix–loop–helix ARNT‐like protein 1
Bv8: Prokineticin 2
CCDC25: Coiled‐coil domain containing 25
cfDNA: Cell‐free DNA
CHT: Caudal haematopoietic tissue
CMP: Common myeloid progenitor
CSF1: Colony‐stimulating factor 1
CTC: Circulating tumour cell
CXCL: C‐X‐C motif chemokine ligand
CXCR: C‐X‐C motif chemokine receptor
EG: Emergency granulopoiesis
EGF: Epidermal growth factor
EMH: Extramedullary haematopoiesis
EMT: Epithelial‐to‐mesenchymal transition
GATA1: GATA binding protein 1
G‐CSF: Granulocyte colony‐stimulating factor
GFI1: Growth factor independent 1
GM‐CSF: Granulocyte–macrophage colony‐stimulating factor
GMP: Granulocyte–monocyte progenitor
HGF: Hepatocyte growth factor
HIF‐1: Hypoxia‐inducible factor 1
HMGB1: High mobility group box 1
HSC: Haematopoietic stem cell
HSPC: Haematopoietic stem and progenitor cell
ICI: Immune checkpoint inhibitor
IFN: Interferon
IFNγ: Interferon gamma
IL: Interleukin
IL‐1RA: Interleukin‐1 receptor antagonist
ILK: Integrin‐linked kinase
Ly6G: Lymphocyte antigen 6 complex locus G
MDSC: Myeloid‐derived suppressor cell
MET: MET Proto‐Oncogene, Receptor Tyrosine Kinase
MMP9: Matrix metalloproteinase 9
MPO: Myeloperoxidase
NE: Neutrophil elastase
NET: Neutrophil extracellular trap
NLR: Neutrophil‐to‐lymphocyte ratio
NO: Nitric oxide
PAD4: Peptidyl arginine deiminase 4
PAMP: Pathogen‐associated molecular pattern
PB: Peripheral blood
PDGF: Platelet‐derived growth factor
PD‐L1: Programmed death‐ligand 1
PGE_2_
: Prostaglandin E_2_
PS: Phosphatidylserine
PU.1 (SPI1): Spleen focus forming virus proviral integration oncogene
RB1: Retinoblastoma 1
RNS: Reactive nitrogen species
RORC1: RAR‐related orphan receptor C1
ROS: Reactive oxygen species
SiglecF: Sialic acid‐binding immunoglobulin‐like lectin F
SNAI1: Snail family transcriptional repressor 1
SOX2: SRY‐box transcription factor 2
STAT3: Signal transducer and activator of transcription 3
TAN: Tumour‐associated neutrophil
TGF‐β: Transforming growth factor beta
Th17: T helper 17 cell
TNF: Tumour necrosis factor
TRAIL: TNF‐related apoptosis‐inducing ligand
TRPM2: Transient receptor potential cation channel, subfamily M, member 2
VEGF: Vascular endothelial growth factor

## 1 Introduction

Neutrophils are the most abundant circulating leukocytes, with an estimated daily production of ~10^10^ cells in humans [1, 2]. Rapidly recruited to sites of infection or injury, they are key players for host defence through phagocytosis, degranulation and the release of neutrophil extracellular traps (NETs) [3, 4, 5, 6, 7]. Beyond these classical roles in acute inflammation, neutrophils display striking phenotypic plasticity, functionally adapting to microenvironmental cues [8, 9]. This versatility enables them to contribute to tissue remodelling and inflammation resolution during physiological conditions, as well as to become involved in disease pathology [10, 11, 12, 13, 14]. While long viewed as primary defender cells, neutrophil plasticity renders them susceptible to hijacking in pathological contexts such as tumorigenesis. The relationship between neutrophils and cancer cells is highly contextual, shaped by host health, systemic influences and tumour characteristics [13, 15, 16, 17, 18, 19, 20].

Within tumours, neutrophils (tumour‐associated neutrophils, TANs) can adopt pro or antitumour functions, shaped by local and systemic signals [16, 17, 18, 19, 21, 22]. A central question is whether tumour‐promoting traits emerge only within the tumour microenvironment or are programmed earlier during neutrophil development. Increasing evidence supports the latter: tumours can remotely influence haematopoiesis, biasing progenitor output towards the granulocytic lineage [23, 24, 25, 26]. This tumour‐induced granulopoiesis generates a chronic supply of immature neutrophils with immunosuppressive and prometastatic properties [27]. Model organisms, notably zebrafish, have been instrumental in visualising neutrophil plasticity and behaviour during tumour initiation *in vivo*, revealing conserved mechanisms that resemble neutrophil phenotypic alterations in advanced tumours. [28, 29, 30].

Here, we review current knowledge on neutrophil biology in the context of tumour development, emphasising the emerging paradigm of tumour involvement in neutrophil ontogeny. We discuss the molecular and cellular mechanisms underpinning neutrophil recruitment and functional heterogeneity and explore how these insights inform the development of neutrophil‐targeted strategies to prevent or delay tumour progression. Finally, we consider how a deeper understanding of neutrophil ontogeny in cancer may offer therapeutic opportunities to normalise myelopoiesis and restore effective antitumour immunity.

## 2 Neutrophil maturation and developmental origins

To understand neutrophil functions within tumours, it is essential to consider their developmental trajectories and maturation programmes. Neutrophil ontogeny is shaped by intrinsic transcriptional networks and extrinsic environmental cues, with distinct programmes under homeostatic versus stress conditions [11, 31, 32].

### 2.1 Steady‐state granulopoiesis

**Fig. 1:**
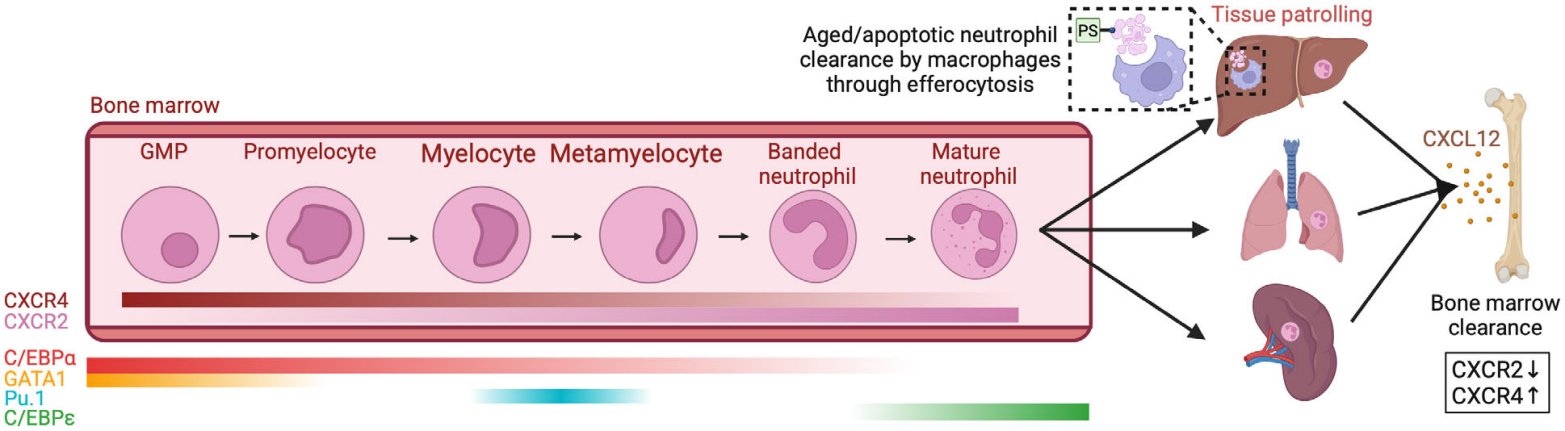
Steady‐state granulopoiesis. Granulocyte–monocyte progenitors (GMPs) in the bone marrow, progressing through sequential maturation stages: promyelocyte, myelocyte, metamyelocyte, banded neutrophil, and mature neutrophil. Stage‐specific regulators: C/EBPα, GATA1, PU.1, and C/EBPε, guide lineage commitment, differentiation, and terminal maturation. During maturation, neutrophils downregulate CXCR4 and upregulate CXCR2, enabling their release from the bone marrow into circulation. Once in peripheral tissues, neutrophils perform homeostatic immune surveillance. As neutrophils age, CXCR2 expression declines while CXCR4 is re‐expressed, promoting homing back to the bone marrow in response to stromal‐derived CXCL12. Senescent or apoptotic neutrophils are then cleared either by resident macrophages in tissues—driven by exposure of phosphatidylserine (PS)—or by bone marrow phagocytes, thereby maintaining systemic neutrophil balance and preventing excessive inflammation.

In mammals, steady‐state granulopoiesis occurs primarily in the bone marrow (BM), while in zebrafish larvae, it arises in the caudal haematopoietic tissue (CHT) [33, 34, 35]. This tightly regulated process ensures a continuous supply of mature neutrophils for blood and tissue surveillance [10]. Haematopoietic stem cells (HSCs) give rise to common myeloid progenitors (CMPs), which differentiate into granulocyte‐macrophage progenitors (GMPs) [36, 37]. Neutrophil maturation proceeds through myeloblasts, promyelocytes, myelocytes, metamyelocytes, band cells and finally segmented neutrophils, with each stage marked by changes in morphology, surface marker expression, granule content and function diversification through tissue environment adaptation (Fig. 1) [38, 39, 40, 41]. In zebrafish larvae, similar lineage trajectories exist, with HSC‐independent waves of neutrophil development arising from primitive myeloid progenitors and a definitive wave producing long‐term HSC‐derived neutrophils later in development [42, 43, 44].

Key transcription factors regulate this differentiation cascade. CCAAT/enhancer‐binding protein alpha (C/EBPα) drives early myeloid lineage commitment and terminal neutrophil differentiation [45, 46, 47, 48, 49], promoting cell cycle exit and effector gene activation. The expression of C/EBPα is high in early progenitors but declines during terminal differentiation [50]. Additional regulators include PU.1 (SPI1), GFI1, LEF1, C/EBPε and RUNX1 further coordinate lineage fidelity and maturation timing [50, 51, 52, 53, 54, 55].

During steady state, neutrophils are released into the circulation as terminally differentiated postmitotic cells, controlled by opposing CXCR2 and CXCR4 signalling [56]. CXCR2, via CXCL1/CXCL2, promotes neutrophil mobilisation from the BM, whereas CXCR4/CXCL12 signalling retains neutrophils in the BM [56, 57, 58]. BM stromal derived CXCL12 is circadian regulated [56]. A diurnal reduction in CXCL12 expression, combined with upregulation of CXCR2 expression in late‐stage neutrophil precursors, facilitates rhythmic BM egress [59, 60]. CXCR4‐deficient neutrophils lose circadian rhythmicity and have reduced BM retention [59]. Circadian oscillations, regulated by core clock gene *Arntl* (BMAL1), continue to shape neutrophil phenotype in circulation [59, 61]. In zebrafish, light‐entrained clock components, Per2 and Cry1a, regulate Reative Oxygen Species (ROS) generation and hmgb1a transcription, enhancing daytime bactericidal activity [62]. Disruption of CXCR2 and CXCR4 balance, or interference with the neutrophil circadian clock, can alter both their regulated release and their functional state in peripheral tissues [63].

Coordinated neutrophil egress from the BM ensures the continuous replenishment of circulating pools and synchronises their functional output with environmental demands. This process is further coupled with neutrophil tissue retention and clearance to prevent excessive accumulation and bystander tissue damage [60]. Neutrophils enter peripheral tissues and undergo time‐dependent ageing, after which macrophages engulf aged neutrophils through efferocytosis, driven by exposure of phosphatidylserine (PS) [10, 64]. In addition to local clearance, a significant portion of aged neutrophils return to the BM for clearance via the CXCR4‐CXCL12 pathway (Fig. 1) [65]. This feedback loop both disposes of aged neutrophils and fine‐tunes granulopoiesis to maintain homeostasis.

While traditionally considered uniform, recent studies have revealed substantial heterogeneity within the neutrophil pool, even under steady‐state conditions [38, 66, 67]. Variations in gene expression, life span, trafficking capacity, cytokine responsiveness indicate that not all mature neutrophils are functionally equivalent, setting the stage for even greater diversity under inflammatory or tumourigenic stress [38, 66, 68].

### 2.2 Emergency granulopoiesis

**Fig. 2:**
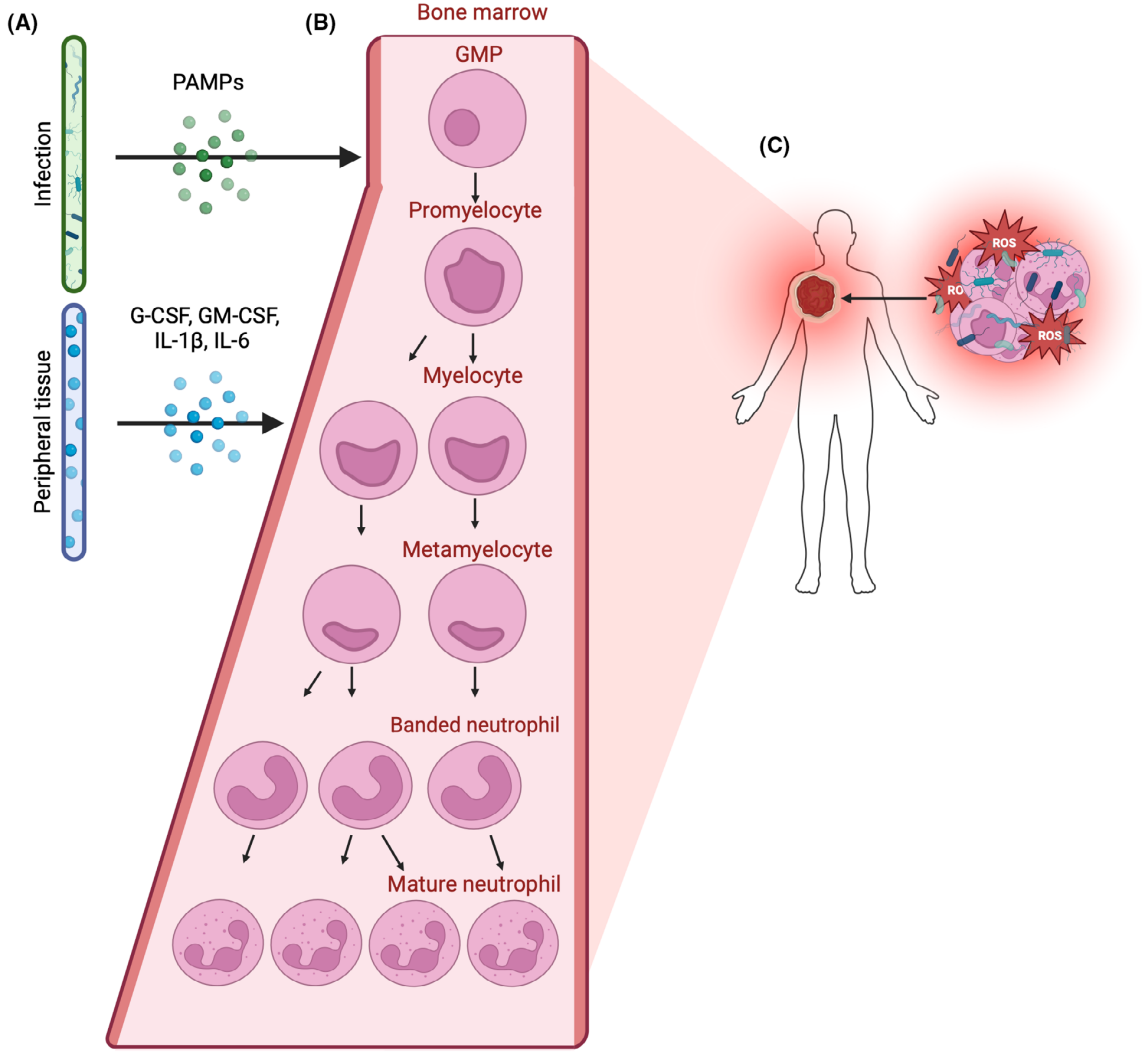
Emergency granulopoiesis (EG). (A) Emergency granulopoiesis (EG) can be initiated through two routes: direct activation, where pathogen‐associated molecular patterns (PAMPs) are sensed by pattern‐recognition receptors on bone marrow progenitors, and indirect activation, where peripheral tissues respond to infection or injury by producing cytokines such as G‐CSF, GM‐CSF, IL‐1β and IL‐6 that signal back to the bone marrow. (B) These cues accelerate granulocyte–monocyte progenitor (GMP) differentiation, leading to enhanced proliferation and premature release of immature neutrophil subsets into circulation. (C) Neutrophils generated under EG are functionally distinct from steady‐state cells, displaying heightened responsiveness and inflammatory activity, including increased production of reactive oxygen species (ROS) that contribute to both host defence [36, 70].

In contrast to the steady trickle of neutrophils produced in homeostasis, emergency granulopoiesis (EG) is a rapid and adaptive ‘surge capacity’ to systemic stressors such as infection, tissue injury or chronic inflammation as seen in cancer (Fig. 2A). During EG, the BM accelerates neutrophil output, not only by expanding myeloid progenitors but also by diverting their differentiation and releasing precursors earlier than usual [69, 70]. Consequently, neutrophil outputs shift towards a heterogeneous population of more immature, transcriptionally distinct and functionally reprogrammed cells compared to their steady‐state counterparts (Fig. 2B) [36, 71, 72, 73].

A centre player in EG is the transcription factor C/EBPβ, which enhances proliferative capacity and skews differentiation towards emergency‐responsive neutrophils [36, 74, 75]. These neutrophils often display a more ‘primed’ phenotype, with heightened responsiveness to inflammatory stimuli, altered surface marker expression and migratory behaviour [70, 72]. The cytokine G‐CSF is the main trigger activating C/EBPβ through Stat3 to stimulate both progenitor expansion and neutrophil egress [71, 72, 76, 77, 78], while other inflammatory mediators, including Il‐6 and IL‐1β, can amplify the response [72, 79].

Collectively, these signals remodel the transcriptional and epigenetic landscape of progenitors, prioritising cell cycle progression, metabolism and antimicrobial function to meet the heightened immune demand [41, 70, 72]. EG neutrophils from sepsis patients and those induced by G‐CSF are immune suppressive, show reduced ability for ROS generation and NETs formation (Fig. 2C) [71, 72, 80, 81, 82, 83]. Thus, the functional features of EG generated immature neutrophils may contribute to their pathological roles [71, 72].

## 3 The dual role of neutrophils in cancer

**Fig. 3:**
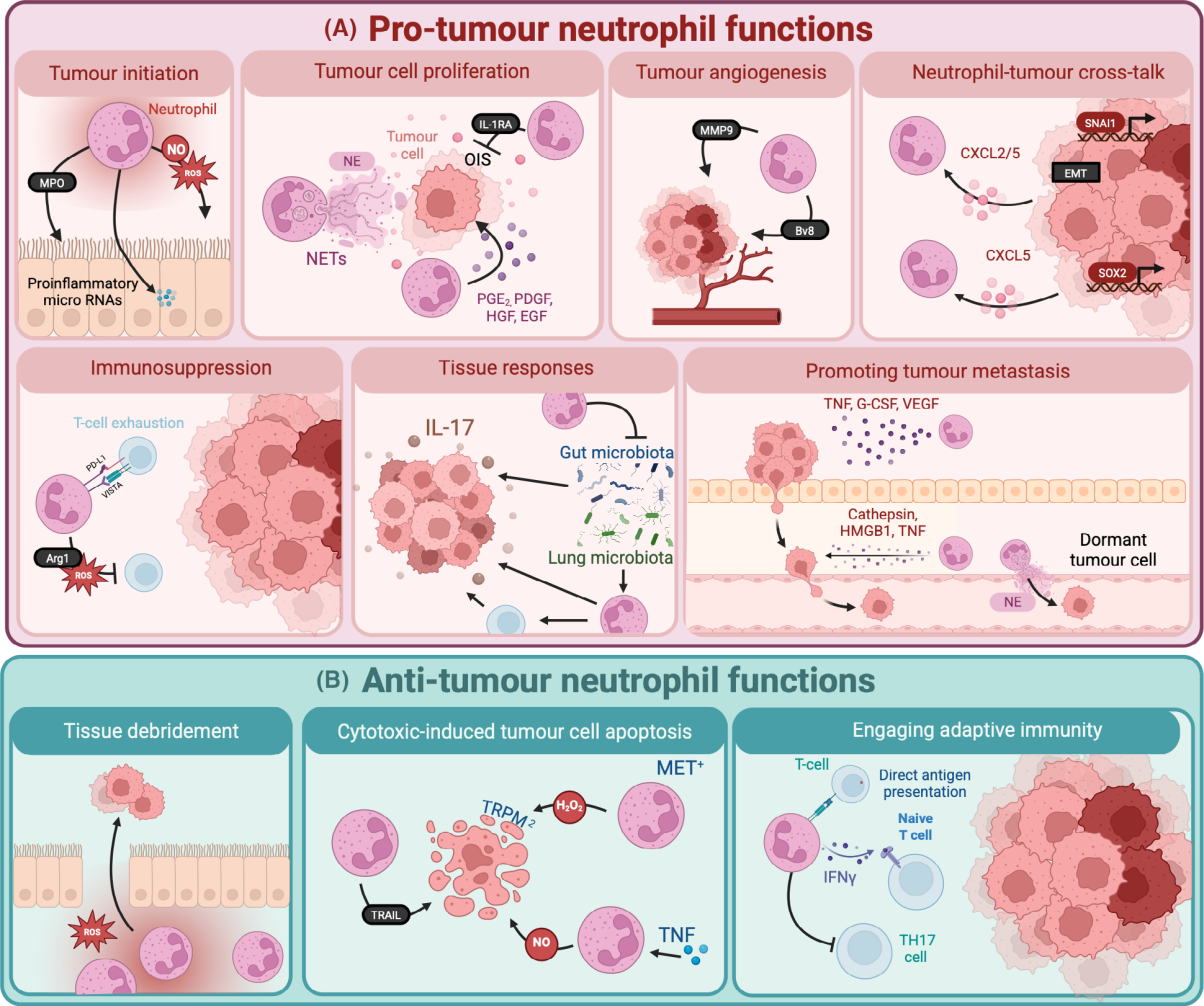
Context‐dependent pro‐ and antitumour functions of neutrophils. (A) Protumour functions include: (i) initiation of tumourigenesis via ROS, MPO, nitric oxide, and pro‐inflammatory microRNAs [140, 223]; (ii) stimulation of tumour cell proliferation through neutrophil elastase (NE), NET formation, and growth factors such as PGE₂, PDGF, HGF, and EGF [224]; (iii) promotion of angiogenesis via MMP9 and Bv8 [225]; (iv) reciprocal cross‐talk with tumour cells, where chemokines (CXCL2/5) and transcriptional regulators (SNAI1, SOX2) reinforce epithelial–mesenchymal transition (EMT) [226, 227]; (v) immunosuppression through arginase‐1, ROS, and PD‐L1–driven T‐cell exhaustion [129]; (vi) modulation of tissue responses via IL‐17 and interactions with the microbiota [228]; and (vii) facilitation of metastatic spread through NE, TNF, HMGB1, cathepsins and premetastatic niche formation [115]. (B) Antitumour functions include: (i) tissue debridement and clearance of damaged cells via ROS; (ii) induction of tumour cell apoptosis through cytotoxic mediators such as TRAIL, nitric oxide, H_2_O_2_, TNF, and TRPM2 activation; and (iii) engagement of adaptive immunity via direct antigen presentation to naïve T cells, secretion of IFNγ, and support for Th17 responses. Collectively, these diverse activities underscore neutrophils as central regulators at the crossroads of cancer progression and immune defence.

Clinical studies consistently show that TAN infiltration correlates with adverse outcomes across many cancer types [84, 85, 86, 87, 88, 89, 90]. Yet their role in cancer is far from straightforward. Neutrophils can act as tumour accomplices or antagonists, with their behaviours shaped by factors such as their maturation state, activation signals, stage of tumour development, and the surrounding tissue microenvironment (Fig. 3) [13, 17, 18, 19, 91, 92]. The once‐popular N1/N2 dichotomy now seems overly simplistic [13, 93]; single‐cell RNA sequencing has uncovered much greater heterogeneity. In lung cancer, for instance, five transcriptionally distinct neutrophil subsets have been identified, including type I IFN–responsive populations present in both healthy and tumour tissue, as well as tumour‐specific subsets expressing CCL3 and CSF1 [94].

Large‐scale pan‐cancer proteogenomic, single‐cell RNAseq, and spatial transcriptomic studies across cancers confirm this striking heterogeneity within the TME, showing how neutrophil states are shaped by both local tumour context and systemic cues [95, 96, 97, 98, 99, 100, 101, 102]. Some populations, such as IL‐1β/CXCR2‐driven neutrophils, are linked to metastasis [27, 103, 104] while others acquire antigen‐presenting capacity and can stimulate T‐cell responses [16, 97, 105]. Conversely, tumour‐derived signals can reprogram neutrophils into immunosuppressive states [106, 107] or promote neutrophil–tumour interactions that enhance tumour progression [108, 109].

### 3.1 Protumour functions of neutrophils

Neutrophils contribute to every stage of cancer development. During tumour initiation, neutrophil‐derived ROS can induce DNA damage thus accelerating DNA mutagenesis (Fig. 3A) [110, 111, 112, 113]. They also stimulate proliferation through NETs containing mitochondrial DNA or proteases. Neutrophil elastase (NE) and matrix metalloproteinase 9 (MMP9) could remodel the extracellular matrix and, in turn, awaken dormant cancer cells [110, 114, 115, 116]. In zebrafish models of RAS‐driven cancer, neutrophil‐derived mediators such as PGE_2_ directly enhanced proliferation [117, 118]. In a *P* *ten* ^−/−^ driven cancer model, neutrophils were shown to release IL‐1RA that alleviated oncogene‐induced senescence [119].

Beyond tumour initiation, neutrophils are involved in remodelling the tumour microenvironment to sustain malignant progression. DNA released during tumour‐induced NETosis engages tumoural DNA receptor CCDC25, exerting chemotactic effects on neoplastic cells via the ILK‐β‐parvin pathway, towards NET‐enriched tissues [120]. NETs were also found to induce the epithelial‐to‐mesenchymal transition (EMT) of cancer cells in breast and gastric cancer [121, 122]. Additionally, NETs sequester circulating tumour cells (CTCs) to aid their transmigration towards distant metastatic sites, with NET‐associated CEACAM1 and β1 integrins acting as major mediators of tumour cell adhesion [123, 124, 125].

In pancreatic cancer, neutrophils mediate the angiogenic switch through MMP9‐VEGF signalling that enhances endothelial sprouting in a previously quiescent vascular tissue [126, 127]. Tumour‐derived G‐CSF also programmes neutrophils to express Bv8, which promotes angiogenesis independently of VEGF [126, 128]. Neutrophils suppress adaptive immunity by depleting extracellular L‐arginine via arginase‐1 and generating ROS/Reative Nitrogen Species (RNS), thereby impairing T‐cell function through suppressed proliferation, diminished IFNγ secretion, and reduced T‐cell cytotoxic activity, ultimately promoting immune evasion in the TME [129, 130, 131].

Together, these findings underscore that neutrophil function is highly plastic, shaped by tumour‐derived cues, hypoxia and chronic inflammation, with multiple mechanisms converging to promote cancer progression. Although alternative cues can also elicit antitumour functions [132].

### 3.2 Antitumour functions of neutrophils

Although neutrophils are frequently associated with tumour promotion, they can also exert potent antitumour activities, operating through direct cytotoxicity, orchestration of adaptive immunity and production of context‐dependent regulatory signals (Fig. 3B) [13].

Neutrophils directly kill tumour cells through multiple effector mechanisms. In a *Pten*‐deficient uterine tumour model, neutrophil‐derived ROS and proteinases promoted tumour cell detachment from the basement membrane and tumour cell death—a process described as tissue debridement [133]. Similarly, HGF/MET‐driven recruitment enabled neutrophils to exert Nitric Oxide (NO)‐mediated cytotoxicity across tumour models [134]. In melanoma and lung cancer, neutrophil‐derived H_2_O_2_ triggered apoptosis of disseminated tumour cells by activating TRPM2, inducing Ca^2+^ influx, mitochondrial depolarisation and cell death [135]. In breast cancer, neutrophil elastase (NE) selectively cleaved and activated CD95/Fas on tumour cells, initiating apoptosis while sparing normal epithelial cells [136]. Human neutrophils also express TNF‐related apoptosis‐inducing ligand (TRAIL), inducing apoptosis of cancer cells *in vitro*; this activity was markedly enhanced by IFNγ, suggesting that cytokine‐activated neutrophils participate in immune surveillance [137].

Beyond direct killing, neutrophils can enhance anti‐tumour immunity by engaging adaptive immune responses. In early‐stage lung cancer, TANs promoted T‐cell proliferation and IFNγ secretion through direct cell–cell contact and cytokine release [91].

These findings illustrate that neutrophil behaviour is highly context dependent. While hypoxia, chronic inflammation and tumour‐derived signals often skew neutrophils towards tumour‐promoting phenotypes, stromal crosstalk and cytokine stimulation, specific tissue niches can instead activate tumour‐restrictive mechanisms [13].

## 4 The inflammatory tumour microenvironment

### 4.1 Inflammation in the preneoplastic niche

The onset of tumourigenesis requires two steps: the acquisition of genomic mutations or epigenetic changes that disable tumour suppressors or hyperactivate oncogenes, followed by the clonal expansion of these transformed cells [138, 139]. Inflammation contributes to both steps, acting as both a mutagenic driver and a microenvironmental amplifier [140, 141].

Preneoplasia represents the earliest phase of tumourigenesis, characterised by oncogenic mutations in epithelial cells without full malignant transformation [142]. These early lesions, which may remain clinically undetected for extended periods, emerge within a dynamic and interactive preneoplastic niche enriched with active innate immune cells including neutrophils [30, 143]. This niche imposes selective pressure on genetically altered cells and shapes clonal evolution towards malignancy [144].

Zebrafish models allow these processes to be visualised in real time, revealing that sustained or dysregulated neutrophil activity fuels RAS‐driven preneoplastic outgrowth through PGE_2_‐mediated inflammation [30, 118, 145], with preneoplastic cells exhibiting epithelial plasticity and metabolic rewiring during clonal expansion [29, 30, 117, 143, 146]. However, in the context of infection‐driven colitis and colitis‐associated colorectal cancer, genetic depletion of neutrophils (*Csf3r* ^−/−^) in a murine model increased susceptibility, with an adoptive transfer of neutrophils sufficient to suppress tumour formation through IL22‐dependent tissue repair [147]. It would appear that the pro‐tissue repair function of neutrophils can either promote or prevent tumour initiation.

These studies emphasise that tumour initiation and progression are not purely cell‐intrinsic processes of mutational accumulation, but rather tissue‐level events shaped by inflammatory cues and immune–stromal crosstalk [138].

### 4.2 The tumour microenvironment and local neutrophil recruitment

Once preneoplastic lesions progress, tumours develop into primary tumours and, ultimately, result in metastatic dissemination [92]. Tumour cells remodel their microenvironment through reciprocal crosstalk with stromal cells and promote angiogenesis via HIF1‐VEGF and MMPs [148, 149]. Tumour and stromal cell‐derived chemokines, especially CXCL family members, form gradients that recruit neutrophils [150, 151, 152]. Among these, signals through CXCR2 and its ligands (CXCL1/2/5/8) are pivotal: they recruit neutrophils and granulocytic MDSCs (myeloid derived suppressor cells), which fuel angiogenesis, promote invasion, immune suppression and drive metastasis [153, 154, 155, 156, 157, 158, 159, 160]. Tumour‐derived IL‐8 (CXCL8) not only recruits granulocytic MDSCs but also enhances their protumoural activity by inducing NET formation [153, 161]. Activated platelets contribute to neutrophil recruitment and immunosuppressive priming by releasing CXCL5, CXCL7, and TGF‐β [162, 163]. In breast cancer, this establishes premetastatic niches [164].

These insights highlight CXCR2 as a therapeutic target. In lung cancer, pharmacological inhibition of CXCR2 curtailed neutrophil infiltration, enhanced cancer cell senescence and apoptosis and synergised with cisplatin to reduce tumour burden and improve survival [165].

## 5 Systemic neutrophil reprogramming in tumour‐bearing hosts

**Fig. 4:**
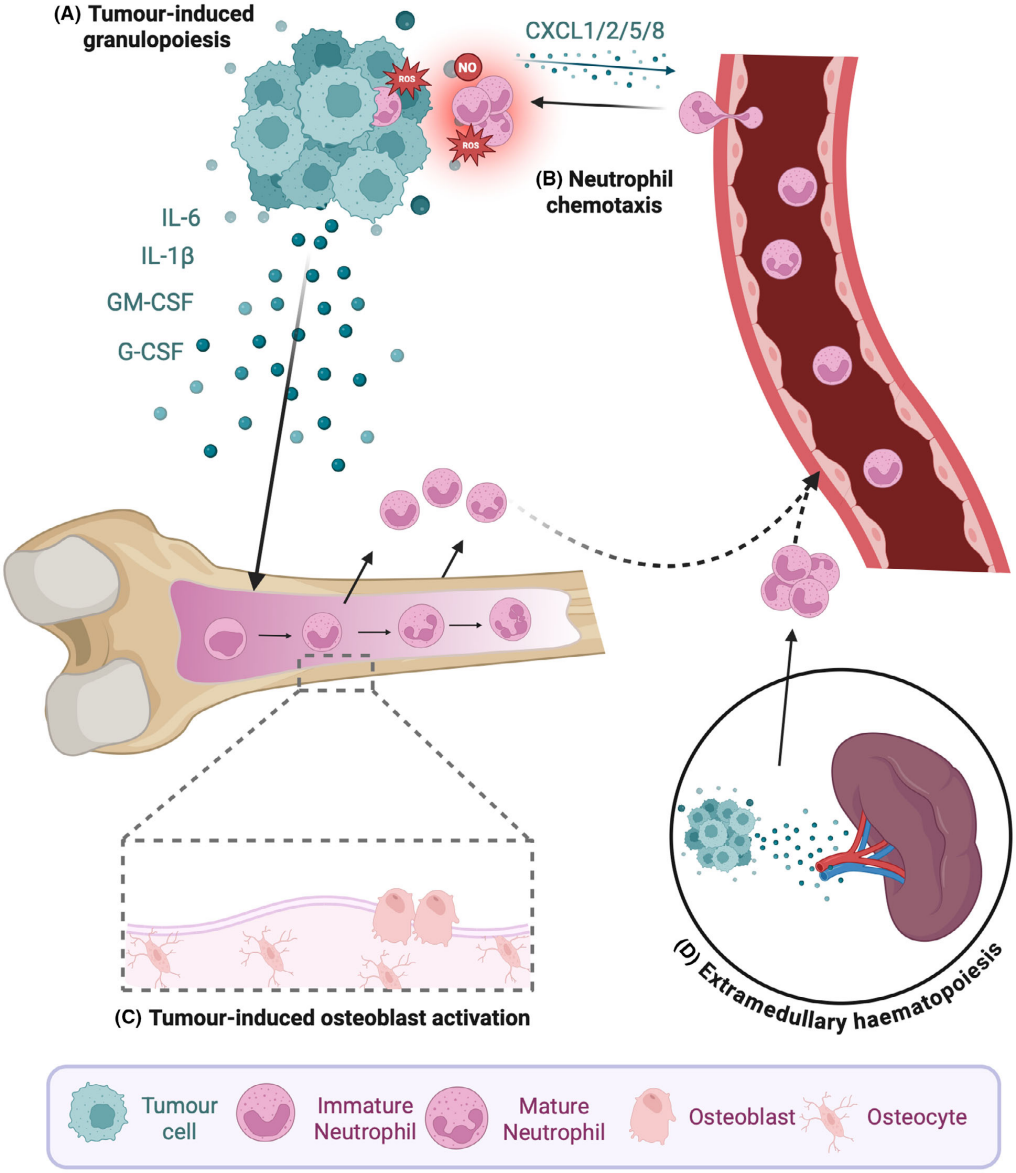
Tumour‐induced granulopoiesis and systemic neutrophil mobilisation. (A) Tumour derived cytokines such as G‐CSF, GM‐CSF, IL‐1β, and IL‐6 drive STAT3–C/EBPβ signalling in progenitors, accelerating granulocytic differentiation and systemic release of immature neutrophils. (B) Tumour‐derived chemokines (CXCL1/2/5/8) establish gradients that recruit neutrophils into the tumour microenvironment, where they promote immunosuppression and support tumour growth. (C) Tumour‐derived factors remotely stimulate osteoblasts in the bone marrow niche, biasing progenitor output towards tumour‐promoting neutrophil subsets (e.g. SiglecF^high^ neutrophils). (D) In addition to the bone marrow, the spleen and other peripheral organs act as reservoirs of tumour‐induced granulopoiesis, supplying further immature neutrophils into circulation.

The prevailing view in tumour neutrophil biology has long been that neutrophils acquire their protumoural functions through local cues within the tumour microenvironment. However, increasing evidence has shown that tumours profoundly reshape early myelopoiesis by skewing haematopoietic output towards granulocyte–monocyte lineages at the expense of erythroid and lymphoid fate [23, 24, 26, 94, 166, 167, 168]. Within this altered developmental landscape, neutrophils egress to an immature and immunosuppressive state, establishing a systemic supply of cells preconditioned to promote tumour growth and immune evasion—a process termed tumour‐induced granulopoiesis (Fig. 4) [167].

Unlike transient infection‐driven emergency granulopoiesis, cancer induced granulopoiesis is chronically sustained by tumour‐ and stroma‐derived cytokines and chemokines, including G‐CSF [26, 169], GM‐CSF [26, 167], IL‐6 [13, 167, 169], IL‐1β [94, 167], TGF‐β [94, 163] and CXCL1/2/5/8 [162, 170], as well as tumour‐derived exosomes (Fig. 4A,B) [166]. This results in persistent elevation of neutrophil production, accumulation of low‐density granulocytes, systemic immunosuppression and poor prognosis for many cancers [26, 167].

### 5.1 Tumour driven reprogramming of myelopoiesis in the bone marrow and periphery

Tumour derived G‐CSF is a key driver that reprograms myeloid progenitors, skewing lineage output towards GMPs via STAT3–C/EBPβ signalling [26, 171]. These progenitors undergo transcriptional reprogramming producing immature neutrophils with enhanced suppressive activity. Early‐stage progenitors with neutrophil‐restricted potential have been identified in both mouse and human BM, where they preferentially give rise to protumoural neutrophils [172]. Recent evidence demonstrates that tumour‐derived G‐CSF induces durable transcriptional and metabolic reprogramming at the progenitor stage, generating functionally impaired neutrophils that persist after G‐CSF withdrawal and confer a sustained susceptibility to bacterial infections in cancer patients [173]. In addition to G‐CSF, IL‐1β plays a central role in tumour‐induced myelopoiesis [174]. In breast cancer, IL‐1β signalling sustains protumoural neutrophil programmes in both primary and metastatic sites [24, 175]. Transcriptomic analyses of BM neutrophils in colorectal cancer‐bearing mice further demonstrated increased IL‐1β expression, with pseudotime inferred trajectory showing altered maturation programmes as neutrophils face premature egress from the BM upon external tumourigenic signals [176]. This establishes tumour‐induced granulopoiesis as a progenitor‐level process rather than a mere by‐product of premature release.

Haematopoietic niche components further reinforce skewed granulopoiesis [23, 25]. Osteoblasts remodel BM niches to supply lung tumours with immunosuppressive SiglecF^high^ neutrophils, whereas osteoprogenitor–GMP crosstalk sustains systemic myeloid bias even after tumour removal (Fig. 4C) [168]. Tumours thus imprint a durable, memory‐like state onto haematopoiesis.

Beyond the BM, extramedullary haematopoiesis (EMH) in the spleen and liver produces immature neutrophils within cytokine‐rich niches, extending the systemic influence of tumours on myelopoiesis (Fig. 4D) [177, 178, 179]. Tumour‐derived cytokines including GM‐CSF, G‐CSF, IL‐3, and IL‐6 mobilise progenitors to splenic red pulp and hepatic sinusoids [180, 181]. In a murine study, hormone‐induced adrenal gland was shown to recruit and host HSPCs via the CXCR4‐CXCL12 axis [182]. Spleen‐derived TAN progenitors have been shown to fuel neutrophil responses, with splenectomy impairing TAN accumulation in lung cancer [183]. Tumour‐induced EMH thus ensures sustained systemic neutrophil supply.

### 5.2 Key signalling pathways and transcriptional drivers that mediate tumour driven granulopoiesis

Tumour‐derived cytokines (G‐CSF, GM‐CSF, IL‐6 and IL‐1β) activate STAT3 and C/EBPβ in progenitors, driving proliferation, accelerated granulocytic differentiation and premature egress [26]. In breast and lung cancer models, RORC1 expression in myeloid progenitors was required for their skewing towards granulocytic/monocytic fates with tumour‐promoting effector functions, including high ROS, arginase‐1, and peroxidase activity [167, 184]. Epigenetic silencing of Retinoblastoma‐1 (RB1) blocks differentiation, allowing immature myeloid cells to accumulate [26, 185], while histamine deficiency impairs maturation, driving CD11b^+^Ly6G^+^ immunosuppressive granulocyte expansion in cancer [186]. Additionally, chemoattractant chemokines such as CXCL1/2 further amplify myelopoietic bias, promoting monocytic MDSC expansion [170]. Collectively, tumours exploit developmental bottlenecks and transcriptional regulators at the progenitor level to enforce suppressive neutrophil states.

## 6 Clinical implications and challenges

Tumour‐induced granulopoiesis represents a systemic dimension of tumour–immune crosstalk, fuelling a continuous supply of neutrophils that are developmentally skewed and functionally reprogrammed to favour tumour progression [26]. By diverting haematopoiesis towards granulocytic and monocytic lineages while impairing lymphoid and erythroid development, tumours drive neutrophilia dominated by immature subsets with potent immunosuppressive and prometastatic activities [32]. Clinically, this manifests as paraneoplastic leukemoid reactions, persistent neutrophilia, and expansion of low‐density granulocytes, features consistently linked to poor prognosis across multiple cancer types [169]. Importantly, such systemic changes may also serve as tractable biomarkers of disease progression and systemic immunosuppression burden [24].

### 6.1 Neutrophil related biomarkers for cancer detection and prognosis

Altered neutrophil production and neutrophil‐to‐lymphocyte ratio have been evaluated as prognosis markers in multiple cancer types [187, 188, 189, 190, 191, 192, 193]. While tissue biopsy has been the standard for molecular analysis and cancer diagnosis, it provides only static information from tumour tissue. In contrast, liquid biopsy approaches are minimally invasive, allow longitudinal sampling, and the ability to capture systemic tumour‐immune dynamics [194]. One such serum‐based neutrophil‐related biomarker is arginase, with elevated arginase levels correlating with advanced disease stage, impaired T‐cell responses, and poor clinical outcomes [195, 196, 197, 198]. Beyond enzymatic markers, NETs provide an additional readout of neutrophil activation and pathological effector function. NETs can be detected in primary tumours, metastatic sites, and the peripheral blood (PB) [199, 200, 201]. As such, studies have shown that changes in tumour‐infiltrated NETs correlate with changes in NETs within the PB [202]. NETs can be detected in the PB through a combination of NET‐related blood products, such as elastase, myeloperoxidase, cell‐free DNA (cfDNA) and citrullinated histone H3 [203, 204, 205]. cfDNA levels in the PB of tumour patients were positively regulated with systemic inflammation parameters, and whose correlation concomitantly increased with tumour clinical stage [203].

### 6.2 Current challenges & approaches in targeting neutrophils therapeutically

One of the principal challenges in targeting neutrophils is the lack of precise markers distinguishing pathogenic from homeostatic subpopulations [206]. Untargeted depletion of neutrophils is impractical given their important role in host defence of infection. TANs may display both pro‐ and antitumour properties, depending on the cytokine milieu. This heterogeneity makes it difficult to selectively inhibit disease‐promoting neutrophils without compromising essential host defence functions. Emerging transcriptomic and proteomic profiling efforts aim to identify neutrophil subsets amenable to targeted modulation [95, 96, 98, 99, 101, 102, 176], yet achieving sufficient specificity without collateral immune suppression remains a major obstacle. Alternative interventions could target the drivers of tumour‐induced granulopoiesis and functional rewiring without ablating normal neutrophil function or targeting cancer‐enriched neutrophil recruitment signals. Type I IFNs induce antitumour polarisation of TANs [207]. Additionally, TGFβ plays a crucial role in driving tumour progression through an immunosuppressive microenvironment, while also driving tumour‐promoting neutrophils. Although neutrophils are not the primary targets, several clinical trials are underway targeting the TGFβ pathway [208, 209].

Blocking neutrophil recruitment through CXCR1/2 antagonism shows promise in reducing neutrophil chemotaxis and immunosuppressive activity without compromising antimicrobial defence in preclinical mouse models [210, 211, 212, 213], a treatment combination of CXCR2 inhibitor (AZD‐5069) with enzalutamide showed partial responses (5/21 patients) in metastatic castration‐resistant prostate cancer [214]. However, early‐phase trials combining CXCR2 antagonists with immune checkpoint inhibitors (ICIs) have demonstrated manageable safety but only limited efficacy so far [215, 216, 217]. Interestingly, in a zebrafish model, cxcr1/2 inhibitors only have limited impact on neutrophil recruitment to preneoplastic lesions and lead to a very small reduction of preneoplastic growth, suggesting additional signals are mediating tumour cell–neutrophil cross talk [30].

Given the pro‐tumour role of NETs, it has been therapeutically targeted through inhibition of PAD4, an enzyme mediating histone citrullination [218]. While multiple reversible and irreversible PAD4 inhibitors are in the preclinical research phase, they face the challenge of low stability and bioavailability [219]. Recent *in vitro* and *in vivo* studies have demonstrated antitumour activity of a novel metal‐based PAD4 inhibitor (BMCIRu) in triple‐negative breast cancer that shows reduced systemic toxicity when compared with traditional chemotherapy agents such as Cisplatin and can be administered orally [220].

An alternative strategy is to intervene earlier in the cascade by targeting tumour‐induced granulopoiesis itself. Prolonged or excessive G‐CSF signalling, while physiologically indispensable, emerges as a key contributor to both the development and activity of immunosuppressive neutrophils in cancer, and the susceptibility of cancer patients to subsequent bacterial infections following treatment [173, 180, 221, 222]. This raises caution about its therapeutic use and positions G‐CSF and GM‐CSF signalling as a critical pathway to modulate rather than eliminate [221]. Targeting upstream inflammatory cues that sustain pathological granulopoiesis offers further opportunities: blocking IL‐1β signalling has been shown to normalise myelopoiesis, restore the generation of functionally competent neutrophils and reduce metastatic burden [24].

## 7 Conclusion

Recognising tumour induced myelopoiesis as a distinct, targetable axis of tumour–host interaction opens new opportunities for therapeutic intervention and biomarker development, with the potential to restore balance in haematopoiesis and re‐enable effective antitumour immunity.

Dysregulated emergency granulopoiesis is a central yet underexplored driver of tumour–immune crosstalk. What begins as a protective host response becomes chronically hijacked in cancer, fuelling the continuous production of immature, immunosuppressive neutrophils that promote tumour growth, angiogenesis and immune evasion. Integrating biomarkers of emergency granulopoiesis—such as circulating G‐CSF, progenitor signatures and neutrophil transcriptional states—into clinical trial design could enable real‐time assessment of therapy‐induced myeloid stress. Therapeutically, combining granulopoiesis modulation with immune checkpoint blockade or anti‐angiogenic strategies offers a rational route to limit suppressive neutrophil replenishment while preserving antimicrobial defence. Reframing emergency granulopoiesis as a tuneable immunological process transforms a hallmark of tumour‐driven inflammation into a tractable target for durable cancer control.

## Conflict of interest

The authors declare no competing interests.

## Author contributions

GM wrote the first draft of the manuscript, edited, and prepared all the figures. YF wrote, reviewed, and edited the manuscript. Both authors reviewed and approved the final version of the manuscript.
